# Fancying the New Rich and Famous? Explicating the Roles of Influencer Content, Credibility, and Parental Mediation in Adolescents’ Parasocial Relationship, Materialism, and Purchase Intentions

**DOI:** 10.3389/fpsyg.2019.02567

**Published:** 2019-11-15

**Authors:** Chen Lou, Hye Kyung Kim

**Affiliations:** ^1^Wee Kim Wee School of Communication and Information, Nanyang Technological University, Singapore, Singapore; ^2^Wee Kim Wee School of Communication and Information, College of Humanities, Arts, and Social Sciences, Nanyang Technological University, Singapore, Singapore

**Keywords:** social media influencers, adolescents, parasocial relationship, materialism, purchase intentions

## Abstract

While social media influencers are gleaning increasing trust and investment from brands, advertisers, and followers, insights on the role of influencers in adolescents’ relationship formation and consumption behaviors are still rare. Drawing on the literatures of influencer content value, influencer credibility, parental mediation, and parasocial relationship (PSR), this study proposed a conceptual model that expounds the appeal of influencers among adolescents. To test the model, we administered an online survey – recruited in proportion to demographic quotas (i.e., age, gender and ethnicity) – among 500 United States adolescents (aged 10- to 19-years old) via Qualtrics panel. Results revealed that, the entertainment value of influencer-generated content, influencer expertise, trustworthiness, attractiveness, and followers’ perceived similarity to their favorite influencers, are positively related to the perceived PSR between adolescent followers and their favorite influencers, which in turn, are associated with adolescents’ materialistic views and purchase intentions. We also explored the role of parental mediation of adolescents’ social media use in their PSR with influencers. Results indicate that, neither active mediation nor restrictive mediation is related to the PSR between adolescents and influencers. Active mediation is negatively associated with adolescents’ materialism, whereas restrictive mediation is positively related to adolescents’ purchase intentions toward influencer-promoted products. This study proposed and tested a comprehensive conceptual model that accounts for the role of influencers in adolescent followers’ materialism and purchase intentions. This study yields three major theoretical contributions. First, it adopts and applies the concept of PSR from the literature of media psychology to explicate influencers’ appeal among adolescents, which lays a theoretical foundation for future research on the impact of influencers. Second, it advances the current literature on social media influencers by specifying key contributing factors for the development of adolescents’ PSR with influencers. Lastly, it explores the roles of the two facets of parental mediation – active and restrictive mediation – in the appeal of influencers among adolescents, which offers directions for future research of parental mediation in the influencer context.

## Introduction

Social media has become a ubiquitous presence in the daily lives of teens and adolescents: with 95% of teens in the United States having access to a smart device and 45% of them reporting that they are constantly connected online (Anderson and Jiang, 2018). Although this young group spends enormous amount of time online and on social media, recent report warns that, a sizable of them – between 36 and 42% of Gen Zers and 31% of teens – hold negative attitudes toward ads and adopt different means to avoid ads (Mediakix, 2018a). One of the most efficient strategies to reach this segment is through influencer marketing, namely brands’ promoting products via “someone like me” (Miachon, 2018). Indeed, 70% of adolescent YouTube users indicated that they treated YouTube influencers as peers and 60% YouTube users would follow influencers’ advice on what to purchase over that of TV or movie celebrities (O’Neil-Hart and Blumenstein, 2016).

Social media influencers are defined as content generators with domain expertise, who can shape followers’ attitudes and purchase decisions (Freberg et al., 2011; Lou and Yuan, 2019). Unlike traditional celebrities’ who gained fame via mass media channels and afford mostly “one-to-all,” non-reciprocal interactions with fans, social media influencers are more like “grass-roots” celebrities who shot to fame via constantly producing valuable content and cultivating reciprocal relationships with their followers via social media (Lou and Yuan, 2019). Accordingly, adolescent social media users treat their relationships with influencers more like friendship rather than fanship (O’Neil-Hart and Blumenstein, 2016).

Recent research on social media influencers have focused on the contributing factors to the effectiveness of influencer marketing (De Veirman et al., 2017), comparing the efficacy of influencers with that of celebrities (Djafarova and Rushworth, 2017), comparing the effectiveness of influencer ads with that of regular ads (Johansen and Guldvik, 2017), the role of disclosure language (Evans et al., 2017, 2018), and proposing a conceptual model that explains the effect of influencers on purchase intentions (Lou and Yuan, 2019). Moreover, research also investigated issues related to the “friendship” between social media personalities/influencers and their followers (e.g., Chen, 2016; Kurtin et al., 2018; Rihl and Wegener, 2019) and identified parasocial relationship (PSR) as an underlying mechanism for endorsement effectiveness or brand-building (e.g., Labrecque, 2014; Chung and Cho, 2017; Kurtin et al., 2018). These studies have offered sufficient evidence on the role of influencer marketing in marketing effectiveness, consumer relationship-building, and/or brand-building. However, few has explored the role of influencers in adolescents’ values and consumption-related behaviors, nor has any offered an overarching framework that unravels the underlying mechanism of influencers’ appeal among adolescents.

Using these studies as a backdrop, the current study seeks to propose a comprehensive conceptual model that explicates the appeal of influencers among adolescents. Specifically, we posit that, three sets of factors – influence content features, influence credibility dimensions, and parental mediation – can shape adolescents’ relationship with influencers and subsequent reactions to influencer-promoted products (e.g., Buijzen and Valkenburg, 2005; Lou and Yuan, 2019). We further identify adolescents’ PSR with influencers as an underlying mechanism through which these factors influence their materialism and purchase intentions.

The findings of this study yield three major theoretical contributions. First, it further applies the concept of PSR from the literature of media psychology to explicate influencers’ appeal among adolescents, which lays a theoretical foundation for future research on the impact of influencers. Second, it advances the current literature on social media influencers by specifying key contributing factors for the development of adolescents’ PSR with influencers. Lastly, it explores the distinctive roles of the two facets of parental mediation – active and restrictive mediation – in forming influencer-adolescent PSR, which offers directions for future research of parental mediation in the influencer context. Practically, findings of this study will inform brands of insights on conducting strategic influencer campaigns targeting adolescents. It also warns parents to be alert to their adolescent child(ren)’s interactions with persuasive online personalities.

## Adolescents and Social Media Influencers

Around 98% of Generation Zers (aged 7–22) own a smart phone. Among them, half of the adolescents spend 10 h or more daily on smart devices (Mediakix, 2018b). As adolescents are spending more time on social media, they are susceptible to the soaring influence of social media influencers. Indeed, influencers’ popularity among these young digital natives has been increasing exponentially in the past few years (Sehl, 2018). For instance, 70% of adolescent YouTube subscribers say they relate to influencers more than to traditional celebrities (O’Neil-Hart and Blumenstein, 2016), and 63% of Generation Zers preferred to see influencers in ads (Mediakix, 2017). One of the reasons for this trend can be that, social media influencers are considered as more “relatable” trendsetters than traditional celebrities, and they can spread advertising messages to the viewers in a more authentic and natural way (Mediakix, 2018b; Lou and Yuan, 2019).

Social media influences and traditional celebrities share some commonality. For instance, both of them enjoy fame and popularity among a sizable number of fans or followers; both can influence fans’/followers’ attitudes and purchases and profit from brand endorsements (e.g., Choi et al., 2005). Unlike traditional celebrities who gained fame through appearing in mass media productions such as TV shows and/or movies, social media influencers cultivate their visibility and popularity via constantly producing valuable content and presenting likable personae on social media (Garcia, 2017). Accordingly, prior researchers termed social media influencers as “a new type of independent third party endorser who shape audience attitudes through blogs, tweets, and the use of other social media” (Freberg et al., 2011, p. 90). Further, Lou and Yuan (2019) defined a social media influencer as “first and foremost a content generator” (p. 59) who attracts substantial number of followers by producing valuable content and profits from promoting sponsored content to his/her followers. Recent research suggests that social media influencers can exert greater influence over adolescents than peers and family members do (Al-Harbi and Al-Harbi, 2017), and that adolescents’ exposure to their favorite media personae on Twitter and interactions with them positively contributed to the strength of their PSR with the media personae (Bond, 2016). During the process of followers’ forming relationship with influencers, influencers’ traits, characteristics of influencer-generated content, as well as the influence of adolescents’ parents, are expected to play indispensable roles (e.g., Schooler et al., 2006; Collier et al., 2016; Lou and Yuan, 2019).

We elaborate on the literatures of influencer content value, influencer credibility, parental mediation, and PSR below to propose the conceptual model.

## Factors in the Conceptual Model

### Influencer Content Value

From the communication exchange perspective, advertising can be viewed as a process of information exchange and relationship building between advertisers and consumers, which can bring value to consumers, help consumers learn about products/brands and thus make informed purchase decisions (Ducoffe and Curlo, 2000). Ducoffe (1995) described advertising value as “a subjective evaluation of the relative worth or utility of advertising to consumers” (p. 1). Advertising that lacks value can inhibit the exchange relationship-building between advertisers and consumers and is likely to lead to consumers’ inattention or even negative ad evaluation, whereas advertising that is high in value is supposed to lead to positive ad attitudes among consumers (Ducoffe and Curlo, 2000). Advertising value consists of three facets: advertising informativeness, entertainment, and irritation (Ducoffe, 1996). Advertising informativeness describes the value of advertising in facilitating informed decisions and subsequent purchase satisfaction. Advertising entertainment value captures advertising’s potential to entertain and to amuse consumers. Advertising irritation refers to advertising’s potential nature of being annoying, offensive, or distracting to consumers, which inhibits consumers from achieving worthy goals. Corresponding with Sun et al. (2010), this study focused on the two positive facets of advertising value – informativeness and entertainment – to quantify the appeal of influencer-generated content.

In applying the conceptualization of advertising value, recent research has explored its role in brand awareness (Dehghani et al., 2016), purchase intentions (Van-Tien Dao et al., 2014), and brand loyalty (Lou et al., 2019). For instance, Lou and Yuan (2019) who explored the role of advertising informativeness value and entertainment value in followers’ trust in sponsored content uploaded by social media influencers revealed that, only informative value of influencer-generated content positively influenced followers’ trust in branded content posted by the influencers.

### Influencer Credibility

Source credibility or endorser credibility is a crucial factor in determining persuasiveness of brand endorsements (e.g., Hovland and Weiss, 1951; Goldsmith et al., 2000; Djafarova and Rushworth, 2017). In influencer marketing, as influencers serve the same roles as celebrities do in brand endorsements, their credibility becomes an important determinant of the efficiency of their endorsements. Prior researchers conceptualized source credibility as a two-dimension construct: expertise and trustworthiness (Hovland et al., 1953). Source expertise captures a source’s qualifications and knowledge to make judgments concerning a certain topic or subject (McCroskey, 1966). Source trustworthiness measures how the message receivers perceive the source in terms of honesty, sincerity, or truthfulness (Giffin, 1967). Further, McGuire (1985) and Ohanian (1990) both conceptualized source attractiveness as a third dimension, which refers to a source’s perceived physical appeal or desirability.

In the celebrity endorsement domain, extant literatures tend to explore the role of endorser credibility in consumer reactions (e.g., Goldsmith et al., 2000; La Ferle and Choi, 2005). More recently, Munnukka et al. (2016) extended the scope of source credibility by including a fourth facet – perceived similarity – when examining peer endorsers. This is more relevant to influencer marketing, as influencers cultivate relationship more like “friendship” with followers and function as peers to followers. Therefore, we adopted the four-dimension of source credibility to gauge the appeal of influencers. Source similarity herein captures followers’ perceived resemblance – including demographic or ideological aspects – between influencers and themselves.

Besides considering the roles of influencer content features and influencer characteristics in PSR, it is noteworthy that relationship formation often happens over a prolonged period of time. When it comes to adolescent’s media use, parents often serve as gatekeeper and mediate their media exposure and activities. Parental mediation of adolescents’ media consumption has been found to mitigate some of the adverse effects of the media on them (e.g., Schooler et al., 2006; Collier et al., 2016). Thus, we introduce a third entity who plays an indispensable role in adolescents’ general socialization with social media – parents (e.g., Fardouly et al., 2018; Ho et al., 2019). We elaborate on the role of parental mediation of adolescents’ social media in the relationship between influencers and adolescent followers below.

### Parental Mediation of Social Media Use

Parental mediation, which is defined as parents’ strategies to control, to monitor and to supervise their children’s media use (Warren, 2001), is considered to help mitigate the negative effects of media use on children’s attitudes and behaviors (Collier et al., 2016). For example, parental mediation has been found to reduce the amount of TV viewing (Schooler et al., 2006) and internet use (Livingstone and Helsper, 2008), which in turn, makes children less susceptible to the negative effects of media exposure on their materialism (Buijzen and Valkenburg, 2005), self-esteem and body image perception (Schooler et al., 2006). Studies that recently examined parental mediation in the context of social media use addressed its antecedents (Krcmar and Cingel, 2016) and its effects on children (Radanielina Hita et al., 2018). Parental mediation of children’s and/or adolescents’ social media use often aims to reduce potential risks involved in the process, including the likelihood to be exposed to child-inappropriate content and susceptibility to cyber bullying and/or privacy invasion (Krcmar and Cingel, 2016).

Besides monitoring children’s media use, co-viewing with children enables parents to mediate the effect of television content (Desmond et al., 1985). However, such media sharing is rare and hard to implement when it comes to social media use that often involves personal devices (Hwang and Jeong, 2015). The current study addresses two commonly examined strategies relevant to parental mediation of adolescents’ social media use: active mediation and restrictive mediation. Active mediation takes place when parents discuss appropriate internet or social media use with their children, whereas restrictive mediation involves parents’ rule setting to control children’s social media use (Krcmar and Cingel, 2016; Symons et al., 2017).

Prior research suggests that active mediation is more effective in reducing the influence of advertising than restrictive mediation (Bijmolt et al., 1998; Buijzen and Valkenburg, 2005). For instance, Bijmolt et al. (1998) found that active mediation cultivates children’s comprehension of adverting, whereas restrictive mediation reduces such comprehension. In a recent study, active parental mediation was effective in reducing the negative impact of social media alcohol ads by enhancing youth’s critical thinking skills (Radanielina Hita et al., 2018). Moreover, active mediation has been found to reduce cyberbullying perpetration and victimization, whereas restrictive mediation was positively related to victimization (Wright and Wachs, 2018). As parents are important socialization agents who can mediate children’s relationships with media characters (Bond and Calvert, 2014; Brunick et al., 2016), parental mediation of adolescents’ social media use is expected to influence their engagement with influencers. However, studies to date have not examined how parental mediation of adolescents’ social media use influences adolescents’ engagement with influencers.

In the proposed conceptual model, we investigate the PSR with influencers as an important mechanism that explains how influencers shape their adolescent followers’ beliefs and behaviors. Specifically, we propose that, adolescents’ interactions with influencers whom they are following, parental mediation of their social media, as well as their engagement with influencer content, jointly contribute to their relationship with the influencers, which in turn, correlates with their materialistic beliefs and consumption behaviors.

### Parasocial Interaction and Parasocial Relationship With Influencers

Parasocial interaction (PSI) describes audiences’ illusory and involved social experiences with media personae (Horton and Wohl, 1956). Audiences often know the media personae well whereas the latter has little knowledge about the former. PSI has often been interpreted as a one-sided and non-reciprocal relationship between audiences and media personae. Although PSI has been mostly studied in the TV context (e.g., Auter, 1992; Russell et al., 2006), recent research extends the application of PSI to the interactive social media context (e.g., Thorson and Rodgers, 2006; Colliander and Dahlén, 2011; Tsai and Men, 2013; Clark, 2015; Chen, 2016; Kurtin et al., 2018; Rihl and Wegener, 2019). PSI has been introduced as an individual’s “interpersonal involvement with a media personality through mediated communication” (Tsai and Men, 2013, p. 78). For instance, Colliander and Dahlén (2011) argued that readers can generate stronger PSI with bloggers through reviewing bloggers’ disclosures of personal life and observing bloggers interact with other readers. Similarly, since social media users not only can “follow” media personae’s updates and observe how they interact with other followers, but also can respond to media personae’s messages, Tsai and Men (2013) argued that social media facilitates a higher level of PSI between the users and media personae.

PSI and PSR have been used interchangeably in some studies (e.g., Kim and Song, 2016; Escalas and Bettman, 2017). However, PSI describes viewers’ short-time relationship perception that is limited to one episode of media viewing or exposure, PSR refers to “a more enduring relationship that a media user forms with a mediated performer” (Dibble et al., 2016, p. 21). PSR thus signals more enduring feelings of “connectedness that audiences have with media personae beyond momentary exposure” (Bond, 2018, p. 459). PSR applies well to the case of influencer-follower relationship. As social media influencers afford reciprocal relationships via regularly generating content and interacting with their followers, followers can develop lasting socioemotional attachment to the social media influencers (e.g., Bond, 2016; Chen, 2016; Kurtin et al., 2018). Specifically, the PSR has been found to mediate the relationship between consumers’ social media interactions and endorser effectiveness (Chung and Cho, 2017), between users’ exposure to YouTube and relationship with YouTube influencers (Kurtin et al., 2018), and between consumers’ brand engagement and brand loyalty (Labrecque, 2014).

Media psychologists have long documented entertainment and cognitive learning as key gratifications people seek from traditional media consumption (Rubin, 1983; Rubin and Perse, 1987; Conway and Rubin, 1991). As concerns the social media context, adolescents’ consumption of influencer-generated content is also likely to be driven by their motivation for entertainment and information-seeking. Because an influencer is “first and foremost a content generator” (Lou and Yuan, 2019, p. 59), the characteristics of influencer-generated content, such as its informative value and entertainment value, are likely to be important antecedents in the process of relationship building between influencers and their followers. Therefore, combining the literature on influencer content value, we argue that, the informative value and entertainment value of influencer content serve as the means/ground that facilitates the formation of influencer-follower PSR. The following hypotheses are advanced:

**H1**: **a)** The informative value and **b)** entertainment value of influencer-generated content will be positively related to the PSR between influencers and adolescent followers.

At the same time, previous studies often explored how media personae’s characteristics – such as similarity and attractiveness – predict the strength of PSR (e.g., Cohen, 2009; Bond, 2018). Studies have shown that viewers construct stronger PSR with media personae whom they consider to be alike and whom they deem as attractive (e.g., Giles, 2002; Tian and Hoffner, 2010; Bond, 2018). However, regarding the role of the two other dimensions of influencer credibility – expertise and trustworthiness – in the strength of PSR, little is known. The following hypotheses and research questions are proposed:

**H2**: Influencers’ **a)** perceived similarity and **b)** attractiveness will positively correlate with the PSR between influencers and adolescent followers.

**RQ1**: How will **a)** influencer expertise and **b)** perceived trustworthiness correlate with the PSR between influencers and adolescent followers?

Besides considering the roles of influencer features and influencer content value, we also explored how parental mediation affects the PSR between influencers and adolescent followers. Although no study to date has examined the role of parental mediation in adolescents’ PSR with influencers, studies have investigated the role of parents’ involvement in how children form PSR with media characters, broadly (e.g., Bond and Calvert, 2014; Brunick et al., 2016). As children are not skilled in developing social relationships, parental encouragement is thought to strengthen their PSR with media characters (Bond and Calvert, 2014). Meanwhile, parental mediation is likely to serve as an important normative cue, which can shape adolescents’ judgment on whether their activities and interactions with influencers on social media are appropriate or not in a given situation. We thus argue that parental mediation of social media use can influence the formation of PSR between adolescent followers and influencers.

Active mediation is more an accommodative approach that is based on the reciprocal discussion of social media use between parents and children (Shin, 2015). Parents are likely to endorse children’s social media activities when they are engaged in active mediation (Radanielina Hita et al., 2018), thereby facilitating adolescents’ relationship building with social media personae, including influencers. In contrast, restrictive mediation is based on rulemaking and regulations, which not only limits the activities that adolescents can perform on social media but also restrict the amount of time that they can devote to social media in general. Over time, we posit that, restrictive mediation, through limiting adolescents’ exposure to social media content and restricting their activities on social media, will prevent them from investing in stronger PSR with influencers. Thus, we propose different directions of the effects that the two types of parental mediation have on PSR:

**H3a**: Active parental mediation of social media use will be positively related to the PSR between influencers and adolescent followers.

**H3b**: Restrictive parental mediation of social media use will be negatively related to the PSR between influencers and adolescent followers.

Essentially, when it comes to advertisers and brands, what matters most to them is the lucrative marketing value of those who are following influencers. Concerning the role of follower-influencer PSR in adolescents’ behaviors, especially consumption behavior, recent literature revealed that role model exerts influence on adolescents’ materialism and marketplace knowledge (Clark et al., 2001). This study focuses on the role of follower-influencer PSR in adolescents’ materialism and purchase intentions toward influencer-promoted products.

### Materialism and Purchase Intentions

Materialism was defined as “the importance a consumer attaches to worldly possessions. At the highest levels of materialism, such possessions assume a central place in a person’s life and are believed to provide the greatest sources of satisfaction and dissatisfaction” (Belk, 1984, p. 291). Although Belk (1985) pointed out that materialism doesn’t necessarily relate to or led to negative outcomes, studies often focused on its role in undesirable behaviors/outcomes, such as compulsive buying (Islam et al., 2018), intention to buy counterfeit products (Furnham and Valgeirsson, 2007), and decreased level of psychological well-being (Christopher et al., 2009).

Social media use has been found to relate to materialistic views among youth (e.g., Debreceni and Hofmeister-Toth, 2018). Concerns regarding the impact of social media influencers on adolescents’ psychological well-being and materialism have been increasing (Gritters, 2019; Stokel-Walker, 2019), it may be partly due to the fact that social comparison is ubiquitous on social media and it can lead to materialistic views (La Ferle and Chan, 2008). Recent research indeed revealed that social comparison with media celebrities positively correlates with adolescents’ materialism, which in turn, predicts compulsive buying (Islam et al., 2018). During this process, social media use moderated the relationship between social comparison and materialism, with increased social media use resulting in intensified materialism (Islam et al., 2018). In another study, de Rezende Pinto et al. (2017) who studied Brazilian’s adolescents (aged 11–18) demonstrated that, their attraction by celebrities, attitudes to TV ads, the influences of parents, peers, and friends, as well as some sociodemographic factors, jointly affect adolescents’ materialistic views. Similarly, Clark et al. (2001) found that role models such as fathers and favorite athletes had the greatest impact on teenagers’ materialism, with father serving to reduce their materialistic views and athletes driving materialism beliefs.

With regard to social media influencers, we argue that influencers serve as role models to adolescent followers and can stimulate social comparisons at times. Moreover, influencers constantly promote sponsored products to followers, which can also drive followers’ interests in material possessions. We posit that, adolescents’ social comparisons with influencers and the fact that influencers often serve as role models to adolescents can both drive adolescents’ materialistic views (e.g., Clark et al., 2001; Islam et al., 2018), which in turn, can correlate with heightened purchase intentions (Islam et al., 2018). Moreover, research also revealed that PSR between social media users and digital celebrities positively predicts users’ purchase intentions (Hwang and Zhang, 2018). Taken together, the following hypotheses are advanced:

**H4**: Adolescents’ perceived PSR with influencers will be positively related to their materialism.

**H5**: Adolescents’ perceived PSR with influencers will be positively related to their purchase intentions towards influencer-promoted products.

**H6**: Adolescents’ materialism will be positively related to purchase intentions towards influencer-promoted products.

**H7:** Adolescents’ materialism will mediate the relationship between their perceived PSR with influencers and purchase intentions towards influencer-promoted products.

**FIGURE 1:**
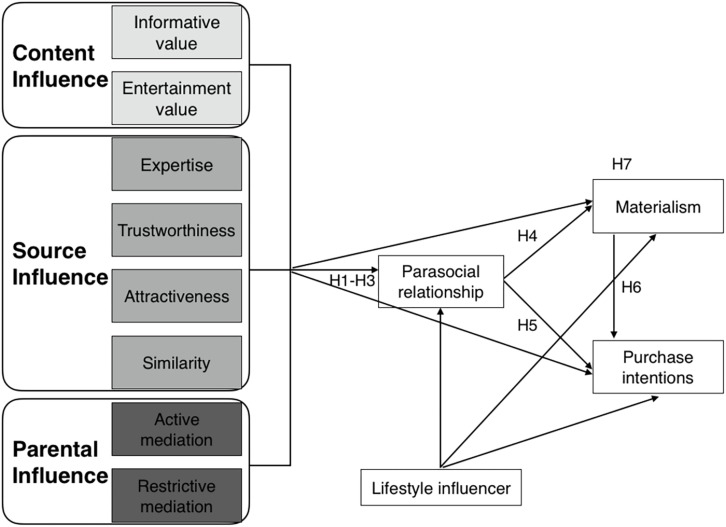
Proposed conceptual model.

A conceptual model that efficiently summarizes this study is proposed (see Figure 1).

## Materials and Methods

### Sample

We recruited adolescents aged 10–19 who are residing in the United States via Qualtrics online panel in July 2019. For those who are 18 and 19, Qualtrics invited qualified panelists to participate in our online survey. Qualtrics invited the parents of those who are under 18 years old and asked the parents to pass the online survey link to their adolescent child/children if the parents consented. For younger adolescents (e.g., aged 10 or 11), we allowed the parents to help their child(ren) understand the questionnaire when necessary, however, parents were instructed not to fill in the survey on behalf of their child(ren). Participants were required to answer the following screening questions asking about (1) their age range, (2) whether the participant is a social media user, (3) whether the participant is following any influencer on social media (the definition of social media influencers is provided along this question, see Appendix), and if so, (3) to write down the name of one social media influencer whom they can recall. Only those aged between 10 and 19 years old who are social media users, who are following influencer(s) on social media and who can name one influencer were eligible to participate. Among those who were eligible, a total of 500 were recruited in proportion to the actual adolescents’ demographic representation (e.g., age, gender and ethnicity) in the United States (we paid $4/per participant to Qualtrics). For example, according to the U.S. Department of Health and Human services (2019), around 50% of United States adolescents age between 10 and 14 years old, and the other half fall in between 15 and19 years old, around 49% of them are females; 55% of them are White, 23% being Hispanic, 14% African Americans, and 5% Asians.

Following the definition of influencers (Lou and Yuan, 2019), online personalities (e.g., YouTuber, Instagrammer, blogger, etc.) who constantly generate valuable contents in a specific domain and who primarily profit from sponsored endorsements are considered influencers. Some reality show celebrity who has a strong online presence and who fits into this definition was also included, including Kylie Jenner^1^. Among the 500 complete responses, those who listed renowned actor/actresses, singers, rappers, soccer players, or politicians (e.g., Trump) as their favorite social media influencer was removed.

A total of 415 responses were entered for data analysis. This pool of participants are 54% females, 53.5% White, with an average of 15 (*SD* = 2.80). 44.6% of them are in high school (9th–12th grader) and the median annual household income falls into the range of $50,000–$74,999. Nearly 89.2% of them have YouTube accounts, with 84.3% having Instagram accounts and 63.9% with Facebook accounts. Around 77.3% of them are following influencers on YouTube, with 65.3% doing so on Instagram and 20.2% on Facebook. As concerns the domains of the influencers, around 39.8% of them are following lifestyle influencers, followed by 31.6% following gaming influencer and 25.3% following fashion influencers (see Table 1 for the demographics).

**TABLE 1:** Demographics of the study sample.

**Measure**	**Items**	**Frequency**	**Percentage**
Gender	Male	185	44.6
	Female	224	54
	Transgender	6	1.4
Race	White	222	53.5
	Black	73	17.6
	Hispanic or Latino	69	16.6
	Asian	33	8
	Other	18	4.3
Education	4^th^ grade	8	1.9
	Middle school (5th–8th grade)	124	29.9
	High school (9th–12th grade)	185	44.6
	College/university	87	21
	Other	11	2.7
Household annual income	<$10,000	48	11.6
	$10,000–$29,999	56	13.5
	$30,000–$49,999	67	16.1
	$50,000–$74,999	78	18.8
	$75,000 or more	78	18.8
	Decline to answer	88	21.2
Type of favorite influencer^∗^	Fashion	105	25.3
	Gaming	131	31.6
	Health living	43	10.4
	Travel	44	10.6
	Lifestyle	165	39.8
	Food	23	5.5
	Pets	15	3.6
	Parenting	20	4.8
	Other	130	31.3
Social media use^∗^	YouTube	370	89.2
	Instagram	350	84.3
	Facebook	265	63.9
	Twitter	196	47.2
	Snapchat	317	76.4
	Other	58	14
Social media used to follower influencers^∗^	YouTube	321	77.3
	Instagram	271	65.3
	Facebook	84	20.2
	Twitter	98	23.6
	Snapchat	97	23.4
	Other	14	3.4

*^∗^Indicates multiple options – “select all that apply,” with total percentage exceeding 100.*

### Procedure

After answering the aforementioned screening questions, qualified participants were directed to read the consent form and to fill in the survey. In the beginning, we included a more detailed definition of social media influencers to help participants understand the questionnaire (see definition in the Appendix). Following the procedure performed by Bond (2018) and Tian and Hoffner (2010), we first asked the participants to report their favorite influencer, and the name given by the participant was inserted in the descriptions of the rest questions (e.g., “Concerning the influencer you have just named – (influencer name), ….” Participants then answered questions asking about their social media usage, perceptions of the content posted by their favorite influencer, influencer credibility, perceived PSR, materialism, purchase intentions, and demographic information. The survey took around 15 min to complete. The participants were debriefed and thanked upon completion of the survey, and they were compensated by the Qualtrics panel. The procedure and instrument of this study have been approved by the Institutional Review Board of the investigators’ institution before data collection.

### Measurement

#### Informative and Entertainment Value

This study measured influencer-generated content’s value by asking the participants to rate influencers’ posts/updates on social media on two sets of 7-point semantic differential scales (Voss et al., 2003), *Ineffective/Effective, Unhelpful/Helpful, Not functional/Functional, Unnecessary/Necessary*, and *Impractical/Practical* for informative value; *Not fun/Fun; Dull/Exciting; Not delightful/Delightful; Not thrilling/Thrilling; and Unenjoyable/Enjoyable* for entertainment value.

#### Influencer Credibility

We measured the four dimensions of influencer credibility by asking the participants to rate their agreement with a series of statements on a 7-point scale (“strongly disagree/strongly agree”) (Munnukka et al., 2016), with statements such as “I feel (influencer name) knows a lot about his/her area” (expertise); “I feel (influencer name) is honest” (trustworthiness); “(influencer name) is good looking” (attractiveness); and “(influencer name) and I have a lot in common” (similarity).

#### Parental Mediation

Parental mediation of social media use was measured through two dimensions – active and restrictive mediation – that were revised based on prior literature (Valkenburg et al., 1999; Ho et al., 2019). Active mediation was measured by four items asking the participants how frequently their parents explained and advised their social media use (“not at all/very frequently,” 7-point scale), with items such as “Telling me to stop any experience on social media if I feel uncomfortable or scared”; Similarly, restrictive mediation was measured by asking the participants how frequently their parents set rules and limitations regarding their social media activities, with items such as “Setting rules regarding my access to social media, such as Facebook, YouTube, Instagram, WhatsApp, etc.”

#### Parasocial Relationship

The strength of PSR was measures by 15 items extracted from Rosaen and Dibble (2016), with items such as “(influencer name) makes me feel comfortable, as if I am with a friend.” Participants indicated their agreement with each of the statements, with options varying from “strongly disagree” to “strongly agree” on a 7-point scale.

#### Purchase Intentions and Materialism

We measured purchase intentions by using three items adopted from Yuan and Jang (2008) on a 7-point scale, including items such as “It’s likely that I would buy certain products/brands because of (influencer name)’s posts.” Participants’ materialism was quantified by four items extracted and revised from Clark et al. (2001) and Gentina et al. (2018), with items such as “It’s important for me to have really nice things” (7-point scale). Table 2 presents the detailed items and their means.

**TABLE 2:** Descriptives of the measurements.

**Constructs (M, SD)**	**Items**	**Means**	**SD**
Informative value (5.76, 1.23)	info_1: ineffective/effective	5.97	1.34
	info_2: unhelpful/helpful	5.78	1.55
	info_3: not functional/functional	5.91	1.38
	info_4: unnecessary/necessary	5.45	1.61
	info_5: impractical/practical	5.69	1.53
Entertainment value (6.37, 1.02)	enter_1: Not fun/fun	6.51	1.11
	enter_2: Dull/exciting	6.39	1.16
	enter_3: Not delightful/delightful	6.35	1.16
	enter_4: Not thrilling/thrilling	6.03	1.46
	enter_5: Unenjoyable/enjoyable	6.58	1.05
Expertise (5.81, 1.36)	expert_1: I feel he/she knows a lot	5.99	1.47
	expert_2: I feel he/she is competent to make assertions about things that he/she is good at	5.82	1.52
	expert_3: I consider he/she as an expert on his/her area	5.66	1.59
	expert_4: I consider he/she sufficiently experienced to make assertions about his/her area	5.78	1.53
Trustworthiness (5.86, 1.41)	trustworthy_1: I feel he/she is honest	5.92	1.51
	trustworthy_2: I consider he/she trustworthy	5.89	1.52
	trustworthy_3: I feel he/she I truthful	5.91	1.52
	trustworthy_4: I consider he/she earnest	5.72	1.52
Attractiveness (5.19, 1.56)	attract_1: He/she is very attractive	5.16	1.82
	attract_2: He/she is very stylish	5.60	1.63
	attract_3: He/she is good looking	5.36	1.71
	attract_4: He/she is sexy	4.64	1.89
Similarity (4.94, 1.55)	similar_1: He/she and I have a lot in common	4.87	1.65
	similar_2: He/she and I are a lot alike	4.74	1.74
	similar_3: I can easily identify with he/she	5.22	1.70
Parasocial relationship (5.83, 1.13)	Parasocial__1: Makes me feel comfortable, as if I am with a friend	5.75	1.49
	Parasocial__2: I look forward to seeing his/her posts	5.74	1.49
	Parasocial__3: I see he/she as a natural, down-to-earth person	5.91	1.46
	Parasocial__4: If he/she starts another social media channel, I will also follow	5.82	1.53
	Parasocial__5: He/she seems to understand the kind of things I want to know	5.59	1.49
	Parasocial__6: If I see a story about he/she in other places, I would read it	5.86	1.45
	Parasocial__7: I would love to meet he/she in person	6.11	1.44
	Parasocial__8: He/she would fit in well with my group of friends	5.47	1.71
	Parasocial__9: If something happens to he/she, I will feel sad	6.01	1.42
	Parasocial__10: I would invite he/she to my party	5.96	1.53
	Parasocial__11: He/she is the kind of persona I would like to play to hand out with	6.00	1.42
	Parasocial__12: If he/she lived in my neighborhood we would be friends	5.72	1.57
Active mediation (5.04, 1.82)	Active_1: Explaining to me the dangers of social media	5.13	2.02
	Active_2: Telling me about the information I can disclose on social media	4.70	2.06
	Active_3: Telling me to stop any experience on social media if I feel uncomfortable or scared	4.96	2.15
	Active_4: Reminding me not out give out personal information on social media	5.37	2.00
Restrictive mediation (3.44, 2.09)	Restrictive__1: Setting rules regarding my access to social media	3.77	2.41
	Restrictive_2: Restricting the amount of time I can use social media	3.43	2.33
	Restrictive_3: Limiting the kind of activities I can do on social media	3.52	2.33
	Restrictive_4: Restricting the type of social media platforms that I can visit	3.52	2.35
	Restrictive_5: Limiting me to using social media only for school work	2.93	2.17
Materialism (5.33, 1.43)	Materialism_1: I would like to be rich enough to buy anything I want	5.70	1.58
	Materialism_2: I’d be happier if I could afford to buy more things	5.15	1.80
	Materialism_3: It sometimes bothers me quite a bit that I can’t afford to buy all the things I want	5.13	1.77
Purchase intentions (5.09, 1.62)	PI_1: Likely to buy certain products because of his/her posts	4.99	1.79
	PI_2: Possible that I will visit some online stores or actual stores because of his/her posts	5.06	1.82
	PI_3: Probable that I may purchase the products/brands that he/she has promoted if I happen to need one	5.22	1.72

### Data Analysis

This study used AMOS 24 to perform both measurement validation, or confirmatory factor analysis (CFA), and structural equation modeling (SEM) testing. All the variables in this study are latent variables with reflective measurements. None of participants’ age, gender, ethnicity, household income, and social media usage was significant in predicting the outcomes and was thus removed from further testing. However, one of the influencers’ domain types – lifestyle influencer – influenced purchase intentions and was thus controlled in model testing. In alignment with the practice of a recent study (Lou and Yuan, 2019), we also explored the direct effects of the predictors on materialism and purchase intentions during model testing.

## Results

### Measurement Validation

A first-order CFA was performed to test the fitness of the measurement model. The model fit for the initial CFA model was not very satisfactory, χ^2^ (1484) = 2879.68, χ^2^/df = 1.94, CFI = 0.92, TLI = 0.91, RMSEA = 0.05, SRMR = 0.06. To boost the model fit, we removed items whose standardized regression weights were below 0.60 (Kline, 2011), including three PSR items and one materialism item. We performed the CFA testing with the revised model: it yielded overall good model fit based on model fit recommendations (Hair, 2010), χ^2^ (1216) = 1930.12, χ^2^/df = 1.59, CFI = 0.96, TLI = 0.95, RMSEA = 0.04, SRMR = 0.05. We examined the standardized loadings, Cronbach’s alphas, composite reliabilities (CR), and average variance extracted (AVE) of the latent constructs to access the reliability and convergent validity of the measurement (see Table 3). Standardized loadings of the latent constructs ranged from 0.66 to 0.94 (Kline, 2011), and their Cronbach’s alphas all exceeded 0.80. The CRs of all latent variables were greater than 0.70 (Hair, 2010). Moreover, all the AVE values of the latent constructs were above 0.50, with the square root of each construct’s AVE greater than its correlation to other latent variables (see Table 4). Therefore, these results indicated that the measurement instrument of this study had sufficient reliability, convergent validity, and discriminant reliability.

**TABLE 3:** Estimates of measurement model.

**Constructs**	**Items**	**Standardized loadings**	**Cronbach’s α**	**CR**	**AVE**
Informative value	info_1	0.84	0.89	0.89	0.62
	info_2	0.78			
	info_3	0.80			
	info_4	0.75			
	info_5	0.76			
Entertainment value	enter_1	0.85	0.91	0.91	0.68
	enter_2	0.82			
	enter_3	0.85			
	enter_4	0.71			
	enter_5	0.88			
Expertise	expert_1	0.89	0.91	0.91	0.73
	expert_2	0.82			
	expert_3	0.84			
	expert_4	0.86			
Trustworthiness	trustworthy_1	0.93	0.95	0.95	0.82
	trustworthy_2	0.90			
	trustworthy_3	0.94			
	trustworthy_4	0.85			
Attractiveness	attract_1	0.94	0.91	0.91	0.73
	attract_2	0.69			
	attract_3	0.93			
	attract_4	0.83			
Similarity	similar_1	0.91	0.90	0.90	0.75
	similar_2	0.87			
	similar_3	0.81			
Parasocial relationship	Parasocial_1	0.78	0.93	0.93	0.52
	Parasocial__2	0.71			
	Parasocial__3	0.70			
	Parasocial__4	0.66			
	Parasocial__5	0.72			
	Parasocial__6	0.74			
	Parasocial__7	0.78			
	Parasocial__8	0.63			
	Parasocial__9	0.71			
	Parasocial__10	0.73			
	Parasocial__11	0.79			
	Parasocial__12	0.72			
Active mediation	Active_1	0.85	0.91	0.91	0.71
	Active_2	0.85			
	Active_3	0.85			
	Active_4	0.83			
Restrictive mediation	Restrictive_1	0.89	0.94	0.94	0.76
	Restrictive_2	0.89			
	Restrictive_3	0.92			
	Restrictive_4	0.93			
	Restrictive_5	0.71			
Materialism	Materialism_1	0.72	0.78	0.78	0.54
	Materialism_2	0.78			
	Materialism_3	0.69			
Purchase intentions	PI_1	0.90	0.89	0.90	0.74
	PI_2	0.82			
	PI_3	0.86			

*CR = composite reliabilities; AVE = average variance extracted.*

**TABLE 4:** Correlations among the latent constructs.

	**1**	**2**	**3**	**4**	**5**	**6**	**7**	**8**	**9**	**10**	**11**
1. Purchase intentions	0.86										
2. Informativeness	0.34	0.79									
3. Entertainment	0.26	0.68	0.82								
4. Expertise	0.35	0.35	0.24	0.85							
5. Similarity	0.44	0.40	0.23	0.44	0.86						
6. Trustworthiness	0.36	0.39	0.28	0.78	0.49	0.91					
7. Attractiveness	0.35	0.21	0.15	0.29	0.33	0.44	0.85				
8. Parasocial relationship	0.55	0.45	0.39	0.55	0.59	0.62	0.48	0.72			
9. Active mediation	0.16	0.17	0.08	0.10	0.30	0.04	–0.17	0.14	0.84		
10. Restrictive mediation	0.18	0.11	0.03	0.06	0.20	–0.06	–0.22	0.06	0.64	0.87	
11. Materialism	0.30	0.19	0.16	0.33	0.15	0.29	0.20	0.35	–0.08	–0.05	0.73

*Diagonal elements are the square root of the AVE for each construct.*

### Structural Model Testing

The proposed model indicated an overall good fit: χ^2^/df = 1.57, CFI = 0.96, NFI = 0.89, TLI = 0.95, RMSEA = 0.04, SRMR = 0.05 (Bentler, 1992; Hu and Bentler, 1999; Tabachnick et al., 2007).

H1 hypothesizes that (a) the informative value and (b) entertainment value of influencer-generated content will be positively related to the PSR between influencer and adolescent followers. Results indicated that informative value was not related to PSR (β = 0.04, *p* = 0.52). However, entertainment value was positively associated with PSR (β = 0.17, *p* < 0.01). Therefore, H1a was not supported, but H1b was supported.

H2 predicts that (a) perceived similarity and (b) attractiveness will be positively related to PSR. Also, RQ1 examines how (a) influencer expertise and (b) trustworthiness will be related to PSR. In support of H2, results revealed that perceived similarity (β = 0.28, *p* < 0.001) and attractiveness (β = 0.24, *p* < 0.001) positively correlated with PSR. Influencer expertise (β = 0.12, *p* = 0.08) and trustworthiness (β = 0.22, *p* < 0.01) were also positively related to PSR.

H3 posits that (a) active parental mediation of adolescents’ social media use is positively related to PSR, whereas (b) restrictive parental mediation is negatively related to PSR. Results indicated that neither active parental mediation (β = 0.08, *p* = 0.15) nor restrictive parental mediation correlated with PSR (β = 0.01, *p* = 0.87). H3 was not supported.

H4 and H5 hypothesizes on the relationships between PSR, materialism, and purchase intentions. In support of H4 and H5, results demonstrated that PSR was positively related to both materialism (β = 0.30, *p*< 0.01) and purchase intentions (β = 0.34, *p*< 0.001). Last, H6 posits that materialism will positively correlate with purchase intentions, and H7 hypothesizes on the mediating role of materialism in the relationship between PSR and purchase intentions. In support of H6 and H7, participants’ materialism was positively related to their purchase intentions (β = 0.14, *p*< 0.05), and materialism mediated the relationship between PSR and purchase intentions (β = 0.05 *p*< 0.05).

**FIGURE 2:**
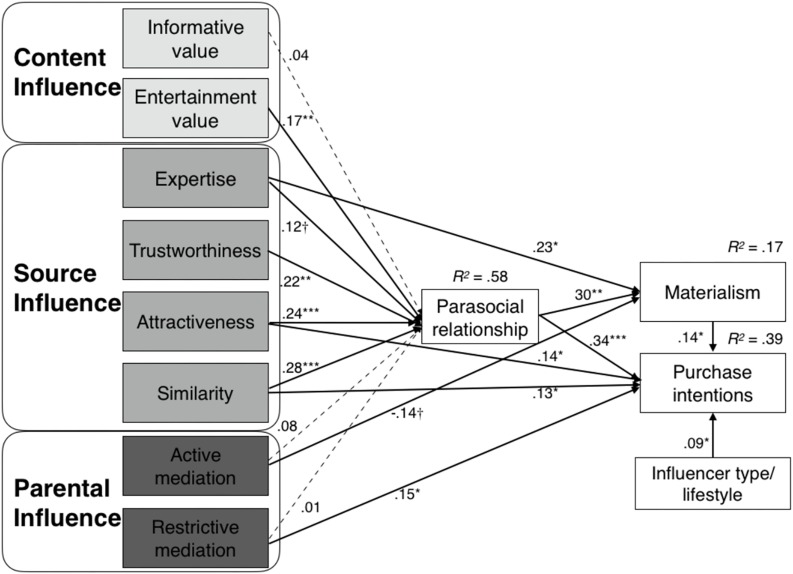
The structural model with path coefficients. Dashed lines indicate non-significant relationships. ^†^*p* < 0.10, ^∗^*p* < 0.05, ^∗∗^*p* < 0.01, ^∗∗∗^*p* < 0.001.

Additionally, we tested the direct effects of the predictors on materialism/purchase intentions. Results indicated that, influencer expertise was positively related to adolescents’ materialism (β = 0.23, *p*< 0.05), whereas active parental mediation was negatively related to materialism (β = −0.14, *p*= 0.07). Influencer attractiveness (β = 0.14, *p*< 0.05), perceived similarity to influencers (β = 0.13, *p*<0.05), and restrictive parental mediation (β = 0.15, *p*< 0.05) significantly correlated with followers’ purchase intentions. Moreover, following influencers who specialize in lifestyle (vs. other domains) is positively related to participants’ purchase intentions (β = 0.09, *p*< 0.05) (see Figure 2). Table 5 summarized the significant direct and indirect effects between the predictors and materialism/PI.

**TABLE 5:** Estimates of the structural model.

**Direct effects**	**Est.**	**SE**	**Std. est.**	
Informative → PSR	0.04	0.06	0.04	
Entertainment → PSR	0.21^∗∗^	0.07	0.17	
Expertise → PSR	0.11^†^	0.06	0.12	
Trustworthiness → PSR	0.19^∗∗^	0.06	0.22	
Attractiveness → PSR	0.16^∗∗∗^	0.03	0.24	
Similarity → PSR	0.22^∗∗∗^	0.04	0.28	
Active mediation → PSR	0.05	0.04	0.08	
Restrictive mediation → PSR	0.01	0.03	0.01	
Influencer type/lifestyle → PSR	–0.07	0.09	–0.03	
PSR → materialism	0.29^∗∗^	0.09	0.30	
Influencer type/lifestyle → materialism	–0.02	0.12	–0.01	
Informative → materialism	0.05	0.09	0.05	
Entertainment → materialism	–0.01	0.10	–0.01	
Expertise → materialism	0.20	0.09^∗^	0.23	
Trustworthiness → materialism	–0.02	0.09	–0.03	
Attractiveness → materialism	0.01	0.05	0.01	
Similarity → materialism	–0.08	0.06	–0.11	
Active mediation → materialism	–0.10	0.05^†^	–0.14	
Restrictive mediation → materialism	0.02	0.04	0.03	
PSR → PI	0.47^∗∗∗^	0.10	0.34	
Materialism → PI	0.19^∗^	0.08	0.14	
Influencer type/lifestyle → PI	0.30^∗^	0.14	0.09	
Informative → PI	0.09	0.10	0.06	
Entertainment → PI	0.03	0.11	0.02	
Expertise → PI	0.09	0.10	0.07	
Trustworthiness → PI	–0.12	0.10	–0.10	
Attractiveness → PI	0.14^∗^	0.05	0.14	
Similarity → PI	0.14^∗^	0.07	0.13	
Active mediation → PI	0.02	0.06	0.02	
Restrictive mediation → PI	0.11^∗^	0.05	0.15	
**Indirect effect via PSR**	**Est.**	**SE**	**Std. est.**	**95%CI**
Informative → materialism	0.01	0.03	0.01	[−0.05, 0.08]
Entertainment → materialism	0.06^∗^	0.04	0.05	[0.01, 0.13]
Expertise → materialism	0.03	0.03	0.04	[−0.01, 0.11]
Trustworthiness → materialism	0.05^∗^	0.03	0.07	[0.01, 0.17]
Attractiveness → materialism	0.05^∗∗^	0.02	0.07	[0.02, 0.15]
Similarity → materialism	0.06^∗∗^	0.03	0.08	[0.03, 0.16]
Active mediation → materialism	0.02	0.01	0.02	[−0.01, 0.08]
Restrictive mediation → materialism	0.00	0.01	0.00	[−0.04, 0.04]
Influencer type/lifestyle → materialism	–0.02	0.03	–0.01	[−0.04, 0.01]
Informative → PI	0.03	0.06	0.02	[−0.05, 0.11]
Entertainment → PI	0.11^∗^	0.06	0.06	[0.01, 0.14]
Expertise → PI	0.09^∗^	0.05	0.08	[0.01, 0.17]
Trustworthiness → PI	0.09^∗^	0.05	0.08	[0.00, 0.18]
Attractiveness → PI	0.09^∗∗∗^	0.03	0.09	[0.04, 0.16]
Similarity → PI	0.10^∗∗^	0.04	0.09	[0.03, 0.18]
Active mediation → PI	0.01	0.03	0.01	[−0.04, 0.07]
Restrictive mediation → PI	0.01	0.02	0.01	[−0.04, 0.06]
Influencer type/lifestyle → PI	–0.04	0.06	–0.01	[−0.05, 02]
**Indirect effect via** **materialism**				
PSR → PI	0.05^∗^	0.03	0.04	[0.01, 0.10]

*PSR = parasocial relationship, PI = purchase intentions; ^†^*p* < 0.10, ^∗^*p* < 0.05, ^∗∗^*p* < 0.01, ^∗∗∗^*p* < 0.001.*

## Discussion

While social media influencers constitute an irreplaceable part of adolescents’ social media use and daily lives, research examining the mechanisms that explain the appeal of influencers among adolescents is sparse. This study advanced the current literature by identifying adolescents’ PSR with influencers as an important psychological mechanism that explains the effect of influencers on adolescents’ materialistic views and purchase intentions. The findings of this study revealed that, the entertainment value of influencer-generated content and influencer credibility – especially attractiveness and similarity – are positively related to the formation of PSR between influencers and followers. Adolescent followers’ PSR with influencers subsequently is positively associated with their materialism and purchase intentions. We elaborated on the major findings of this study as follows.

One major finding pertains to the role of influencer-generated content value, especially entertainment value, in shaping followers’ PSR with influencers. This adds to the literature on the determinants of PSR. Prior literature on the antecedents of PSR often focused on the relationship between media users and media personae (i.e., celebrities or fictional characters) (see a review in Giles, 2002). Recently, researchers extended the application of PSI/PSR to social media contexts (e.g., Colliander and Dahlén, 2011; Clark, 2015; Chen, 2016; Kurtin et al., 2018; Rihl and Wegener, 2019). Taking a step further, this study took the unique status of influencers – content generators – into consideration when examining the contributing factors of PSR. It is interesting that only the entertainment value, but not informative value, of influencer-generated content correlated with followers’ PSR with influencers. Although Lou and Yuan (2019) revealed that the informative value of influencer content positively predicts followers’ trust in influencer’s branded posts whereas entertainment value shows no impact, the findings of this study suggest the other way around. It can be explained that, when followers assess the quality/credibility of influencers’ sponsored posts, informative value is crucial, whereas followers place more emphasis on the entertainment value of influencer content when forming illusory social relationship with their favorite influencer.

The second major finding relates to the role of source credibility dimensions in the process of developing PSR with influencers. The findings of this study advanced extant literature on the roles of attractiveness and similarity in audiences’ PSR with media characters (e.g., Giles, 2002; Tian and Hoffner, 2010; Bond, 2018) by further considering the impacts of expertise and trustworthiness. In agreement with Bond (2018), the findings of this study suggest that, perceived similarity to one’s favorite influencer and attractiveness of the influencer are positively related to PSR. In addition, influencer trustworthiness and expertise were found to be positively related to PSR. Hoffner and Buchanan (2005) once theorized on the roles of TV character attributes (e.g., smart, successful, attractive, funny, violent, admired) in viewers’ wishful identification with them. They reported that both men and women identify more strongly with TV characters of the opposite gender who are successful and admired. Our findings suggest expertise and trustworthiness as important factors that are associated with PSR, and thus contributes to the theory development on the roles of influencer attributes in PSR.

Counter to our expectation, results showed that parental mediation was not related to adolescents’ PSR with influencers. However, active mediation was found to be negatively associated with adolescents’ materialism. Prior research has suggested enhanced critical skills and media literacy as possible mechanisms of how parental mediation mitigate negative media effects (Bijmolt et al., 1998; Radanielina Hita et al., 2018). Thus, it can be explained that, active parental mediation help adolescents develop critical skills to interpret influencer content, which in turn, mitigates their materialism. Meanwhile, our finding suggests that restrictive mediation is not related to PSR, but it is positively associated with adolescents’ purchase intentions. Previous research indicated that, adolescents, especially as they grow older, are less receptive to restrictive parental mediation (Warren et al., 2002; Panek, 2014). This may expound the lack of correlation between restrictive mediation and PSR. Further, we speculate that, adolescents’ psychological reactance, a motivational state to preserve one’s own freedom and autonomy (Brehm and Brehm, 2013), can arise when they are subject to restrictive mediation. The occurrence of reactance may explain the positive relationship between restrictive mediation and purchase intentions. Nonetheless, we acknowledge that these are possible speculations, which needs further validation.

Future work can also explore the roles of alternative parental mediation strategies that are beyond active and restriction mediations, particularly those that are more suitable for adolescents in the social media context (Valkenburg et al., 2013). We also recognize that the current measurement of parental mediation focused on adolescents’ social media use. Given that foreseeably not all parents are aware of the recent phenomenon of influencers, we did not directly examine parental mediation of adolescents’ interactions/relationship with influencers. However, future research needs to narrow down the scope of parental mediation and test its influence on adolescents. As Shin (2015) mentioned that parents of children aged 7–12 had high confidence in their management in relation to their children’s Internet use and were thus less interested in updating their Internet knowledge, we warn parents to be alert to this recent phenomenon – the soaring popularity of social media influencers – and the influencers’ impact on their adolescent followers.

Additionally, the findings of this study pointed to the negative implications of adolescents’ PSR with influencers, such as cultivating adolescents’ materialistic views and shaping their intents to purchase endorsed products. Although Belk (1985) mentioned that materialism doesn’t necessarily lead to negative consequences, findings of this study suggest that it can boost purchase intentions among adolescents and mediates the relationship between PSR and purchase intentions. The finding that materialism serves as an underlying mechanism through which PSR shapes purchase intentions, adds to the extant literature on the antecedents of materialism and its impact on adolescents (e.g., Clark et al., 2001; Islam et al., 2018). Given influencer’s critical role in adolescents’ daily lives, future work is warranted to further investigate the role of adolescents’ PSR with influencers in other related outcomes, for example, psychological well-being and self-esteem.

Lastly, the *post hoc* analysis on direct effects indicate that, influencer expertise was positively associated with adolescents’ materialism, and influencer attractiveness and followers’ perceived similarity to influencers were positively associated with adolescents’ purchase intentions. Previous literature broadly mentioned that social comparison on social media (La Ferle and Chan, 2008) and social comparison with peers/celebrities (Clark et al., 2001; Islam et al., 2018) can drive materialistic views among adolescents. The current finding advanced the literature and indicates that, influencer trait – expertise – can directly shape adolescents’ materialism. Future work needs to further investigate the relationship between influencer characteristics and followers’ materialism. Interestingly, influencer attractiveness and followers’ perceived similarity to influencers are positively related to followers’ purchase intentions. This finding further advanced a recent conceptual model on how influencers affect purchase intentions among adults (Lou and Yuan, 2019).

Practically, influencers are advised to rely on the entertaining value of their content to foster stronger PSR with followers. Specifically, influencers can emphasize on aspects that they have in common with followers and cultivate presentable and attractive online personalities to strength their relationship with followers. Moreover, influencers should also focus on sharing valuable content that signals domain expertise, as well as cultivating their perceived trustworthiness among followers. Given that PSR is positively related to adolescents’ materialism and PI, parents should be aware of the appeal of influencers and engage in active mediation. Influencers, on the other hand, are advised not to overly promote material possessions and/or social comparisons among adolescent followers.

### Limitations and Future Directions

This study also bears its limitations. First, we acknowledge that this study adopted a survey approach and unraveled significant correlations, instead of causal relationships, among the key constructs. Future research should use experimental designs or time series analysis to make causal statements about the hypothesized relationships. Second, this study considers three sets of critical factors – influencer content, influencer credibility, parental mediation – when examining the mechanism that explains the appeal of influencers among adolescents. We acknowledge that there may be other relevant factors (e.g., peer influence, advertising literacy) that were not included in this model. The conceptual model proposed in this study serves as a foundation for more in-depth understanding of the impact of influencers on adolescents. Third, we measured parental mediation of adolescents’ social media use, which may not well capture the roles of parents in regulating adolescents’ engagement with influencers. Future research should aim at proposing and testing new measurement of parental mediation in relation to influencer-adolescent interactions to better examine the role of parental mediation. Last, we included adolescents who aged 10–19 years old in this study and found that age did not play a role in the model testing. Although some researchers considered the role of parental mediation among 18 years old (e.g., Nikken and Jansz, 2006; Shin and Lwin, 2017), it is not very common to examine among those who are 19 years old. Future research needs to further test the age boundary of the impact of parental mediation.

## Conclusion

Just like what media personae did with audiences of tradition media (e.g., TV), social media influencers can foster illusory PSR with followers. However, present-day social media influencers are unlike media personae from earlier days: influencers can engage followers in two-way interactions via social media and foster stronger PSR with followers not only via content generation but also through cultivating “authentic” and desirable personal attributes. Adolescents’ perceived PSR with influencers is positively related to their materialistic views, which in turn, correlates with their purchase intentions toward influencer-promoted products.

## Data Availability Statement

The datasets generated for this study are available on request to the corresponding author.

## Ethics Statement

The studies involving human participants were reviewed and approved by the Nanyang Technological University Institutional Review Board (IRB).

## Author Contributions

CL, as the PI, contributed to the theorization and data collection of this study, who also wrote most of the original manuscript and its revised version. HK assisted in theorization, data collection, and revision, who wrote a literature review section and related discussion parts for both the original manuscript and its revised version.

## Conflict of Interest

The authors declare that the research was conducted in the absence of any commercial or financial relationships that could be construed as a potential conflict of interest.

## Footnotes

1 “Traditional celebrities can also develop some influencer status, but only after they have become regular content creators” (Lou and Yuan, 2019, p. 60). Traditional celebrities/reality stars who are creating content on social media and regularly interact with followers are also considered as influencers.

## Appendix

### Definition of Influencers

Social media influencers are digital personalities who have amassed large number of followers across one or several social media platforms (e.g., YouTube, Instagram, Snapchat, or personal blogs) and carry influence over others. Compared with traditional celebrities, influencers are “regular people” who become online “celebrities” by creating content on social media, e.g., toys review YouTuber *Ryan*, gaming YouTuber *PewDiePie*, Instagram star *Loki the Wolfdog*, fashion influencer *Aimee Song*, among other influencers in areas like toys, gaming, healthy living, travel, lifestyle, food, etc.
